# Human adenovirus type 5 vectors deleted of early region 1 (E1) undergo limited expression of early replicative E2 proteins and DNA replication in non-permissive cells

**DOI:** 10.1371/journal.pone.0181012

**Published:** 2017-07-10

**Authors:** Bratati Saha, Robin J. Parks

**Affiliations:** 1 Regenerative Medicine Program, Ottawa Hospital Research Institute, Ottawa, Ontario, Canada; 2 Department of Biochemistry, Microbiology and Immunology, University of Ottawa, Ottawa, Ontario, Canada; 3 Centre for Neuromuscular Disease, University of Ottawa, Ottawa, Ontario, Canada; 4 Department of Medicine, The Ottawa Hospital, Ottawa, Ontario, Canada; University of St Andrews, UNITED KINGDOM

## Abstract

Adenovirus (Ad) vectors deleted of the early region 1 (E1) are widely used for transgene delivery in preclinical and clinical gene therapy studies. Although proteins encoded within the E1 region are required for efficient virus replication, previous studies have suggested that certain viral or cellular proteins can functionally compensate for E1, leading to expression of the early region 2 (E2)-encoded replicative proteins and subsequent virus replication. We have generated a series of E1-encoding and E1-deficient Ad vectors containing a FLAG-epitope tag on each of the E2-encoded proteins: DNA-binding protein (DBP), terminal protein (TP) and DNA polymerase (Pol). Using these constructs, we show that for the replication-competent virus, the expression level of each E2-encoded protein declines with increasing distance from the E2 promoter, with E2A-encoded DBP expression being ~800-fold higher than E2B-encoded TP. Pol was expressed at extremely low levels in infected cells, and immunoprecipitation from cell lysates was required prior to its detection by immunoblot. We further show that DBP was expressed 200- to 400-fold less efficiently from an E1-deficient virus compared to a replication-competent virus in A549 and HepG2 cells, which was accompanied by a very small increase in genome copy number. For the E1-deficient virus, late gene expression (a marker of virus replication) was only observed at very high multiplicities of infection. These data show that E1-deleted Ad gives rise to limited expression of the E2-encoded genes and replication in infected cells, but highlight the importance of considering viral dose-dependent effects in gene therapy studies.

## Introduction

**Fig 1:**
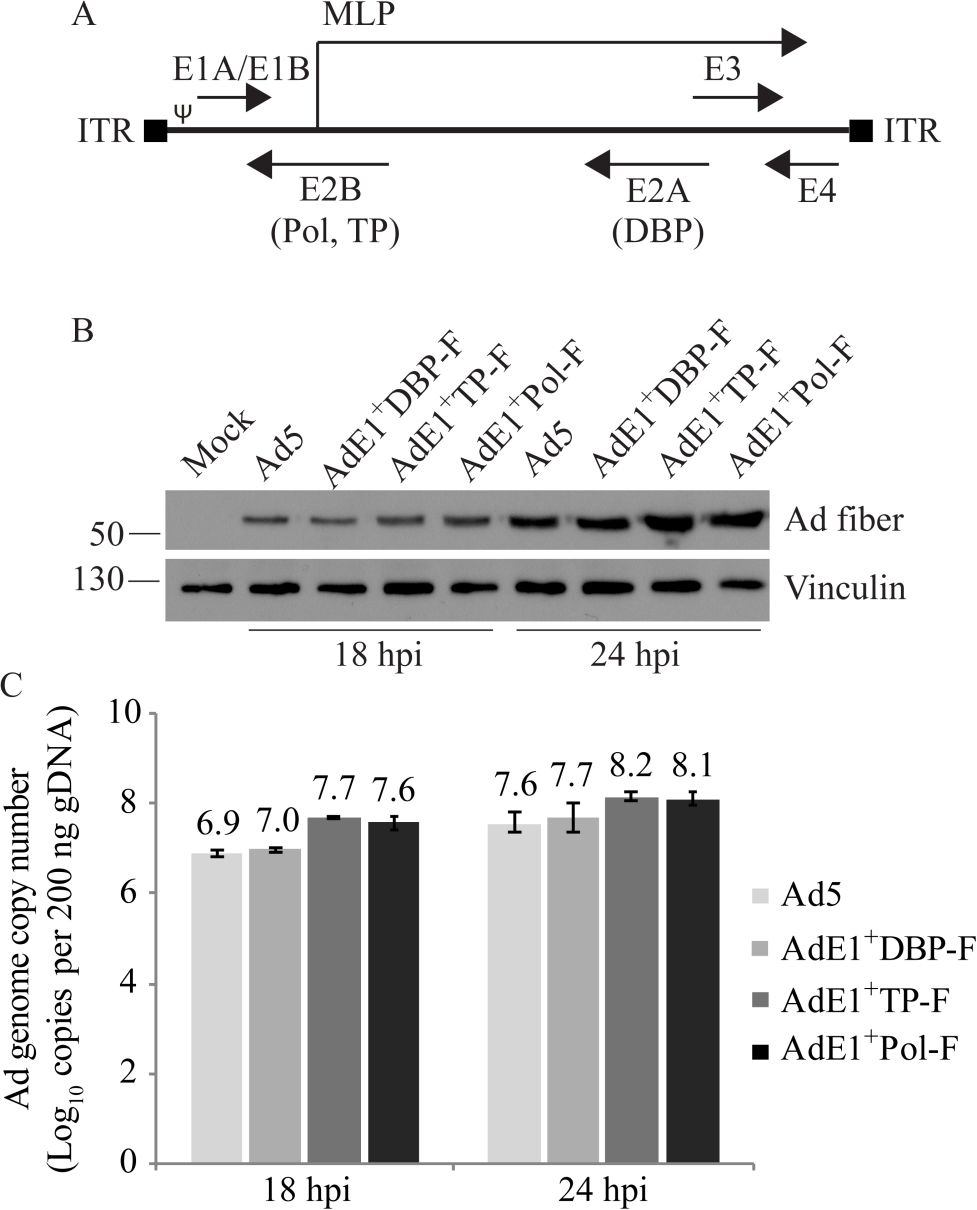
Growth of Ad constructs containing FLAG-tags within the E2 proteins is comparable to wildtype Ad5. (Panel A) Schematic diagram of the wildtype Ad genome (not drawn to scale) showing locations of the early regions E1 to E4. The E2 region consists of E2A and E2B transcription units, which encode for DBP and TP/Pol, respectively. Both E2A and E2B are expressed from a common promoter located upstream of the E2A coding region. MLP is the major late promoter, ITR represents the inverted terminal repeats, and ψ is the packaging element. (B) A549 cells were infected at a MOI of 10 with Ad5, AdE1^+^DBP-F, AdE1^+^TP-F or AdE1^+^Pol-F and 18 or 24 hours post infection (hpi), the cells were lysed for protein and DNA collection. Immunoblot analysis of fiber expression shows no significant difference between the viruses. Vinculin serves as a loading control. (C) Viral DNA copy number per 200 ng of cellular DNA, as determined by qPCR, are also similar for all four viruses at both 18 and 24 hours. Copy numbers were calculated based on a standard curve obtained by serially diluting a plasmid containing the hexon coding region. The mean of three experiments are shown and the error bars represent standard deviation (SD) of the mean.

Human adenovirus (Ad) mainly causes self-limiting respiratory illnesses and can rapidly spread through confined populations such as day care centers, hospitals, retirement homes and military training venues [1]. Since their identification in the 1950’s, over 100 serotypes of Ad have been isolated from a variety of species. Of the human Ads, serotypes 2 and 5 (Ad5) have been extensively studied to gain a better understanding of virus biology, host cellular processes and virus-cell interactions during infection [2]. The Ad5 genome (36 kb, double-stranded DNA) consists of “early” and “late” regions, which are expressed before and after viral DNA replication, respectively (Fig 1A). The early regions (E1A, E1B, E2, E3 and E4) encode proteins that are involved in activating transcription of other viral regions, altering the host cellular environment to enhance virus replication, or in replication of the viral DNA [3]. The E1A gene products are the first proteins expressed from the infecting virus, and these proteins transactivate other viral coding regions, interact with a multitude of cellular proteins, and have a variety of other functions that ultimately promote infection [4]. The major product from the E1B region, the 55-kDa protein, is required for selective export of viral late mRNAs from the host cell nucleus; it also acts (in cooperation with E1A and E4orf6) to induce degradation of certain cellular proteins and promotes transformation of the infected cells [5,6]. Together, the E1A and E1B coding regions are absolutely required for efficient viral gene expression and replication.

The E2 transcription unit consists of two regions, E2A and E2B, which have separate polyadenylation sites [7]. The E2A region encodes the 72-kDa DNA-binding protein (DBP), which plays a crucial role during the elongation phase of Ad DNA replication: DBP multimerizes on the single-stranded DNA (ssDNA) template, and is mainly responsible for unwinding the template [8]. DBP association with ssDNA may also function to protect the viral DNA and prevent immune and DNA damage responses that can be induced in the cell against naked DNA from foreign species [9]. The E2B region codes for the 80-kDa precursor terminal protein (pTP) and the 140-kDa Ad DNA polymerase (Pol), which, along with DBP and other cellular proteins, form a pre-initiation complex prior to Ad DNA replication [10]. Initiation of DNA synthesis occurs via a protein-priming mechanism where Pol covalently attaches a dCMP residue to the pTP in the complex. The 3'-OH group of the dCMP then serves as a primer for elongation by Pol through a strand-displacement process [11]. During virion maturation in the late phases of infection, pTP is cleaved by the Ad-encoded protease to produce the mature 55-kDa terminal protein (TP), which remains associated with each 5’ end of the linear Ad DNA [12]. The E3 and E4 proteins alter host immune responses and cell signaling, respectively [13,14].

In general, the late transcription units, L1-L5, are produced from alternative splicing of a common major late transcript, and code for structural and capsid proteins (e.g. fiber, hexon) required for the production of progeny virions. In addition to these major early and late proteins, four other small products are produced by Ad: protein IX (pIX), virus-associated (VA) RNA I, VA RNA II and the U exon protein (UXP). pIX is a structural protein expressed at an intermediate time during infection (after initiation of early gene expression and before late gene expression), and it plays a role in virion stability [15,16]. VA RNA I and II are double-stranded RNA molecules produced during late infection, and they promote viral protein synthesis [17], inhibit activation of the interferon-mediated antiviral responses [18] and impede cellular micro-RNA processing [19]. UXP, also expressed during late infection from a unique promoter, associates with viral replication centers throughout the host nuclei and has been suggested to play a role in DNA replication or RNA transcription [20,21].

Ad vectors are one of the most commonly used platforms for gene delivery in human gene therapy clinical studies, as well as in biochemical studies conducted *in vitro* [22]. First generation Ad (fgAd) vectors are deleted of the entire E1 region, which is commonly believed to render the virus replication-deficient in most cell lines. These vectors are ideal for studies involving short-term gene expression either *in vitro* or *in vivo* [23]. One major limitation of the fgAd vectors *in vivo* is the transient expression of the therapeutic transgenes due to rapid clearance of the transduced cells, caused in part by induction of immune responses to Ad proteins produced from the viral protein coding regions retained in the vector [24,25]. Furthermore, DNA replication and late gene expression of fgAd has been observed in many “non-permissive” cell lines (*i*.*e*. cells that do not complement the E1A/E1B deletion in these vectors) at multiplicities of infection (MOI) as low as 10 [26]. In such cases, it has been speculated that activation of the E2 promoter by viral or cellular proteins and subsequent expression of the E2 replicative proteins likely led to the observed vector replication [27,28]. For example, the Ad E4-ORF6/7 fusion protein can transactivate the E2 promoter to a considerable degree, especially in the absence of E1A [29,30,31]. Consequently, E4-ORF6/7-mediated activation of E2 expression has been implicated in partially circumventing the replication-defective nature of E1A-deleted vectors used in various gene therapy applications [31], allowing fgAd to give rise to virus replication at high levels in some cell lines [26,27].

Although antibodies against the E2-encoded proteins have been generated by some research laboratories, they are not commercially available or readily accessible [32,33,34]. Thus, to facilitate studies of these proteins, we have inserted a FLAG-epitope tag individually into Ad5 DBP, TP and Pol. We show that the presence of the tag does not adversely affect virus replication, and that each protein could be easily detected by immunoblot of infected cell lysates or virions. We also examined the relative levels of DBP expression and DNA replication from an E1-deleted virus in cell lines that do not complement the E1-deletion. Although E2 proteins were expressed at low levels, removal of E1 severely compromised the ability of the virus to replicate.

## Materials and methods

### Cloning FLAG-tags into the E2 region of Ad5

All Ad constructs were cloned using a combination of conventional and bacterial RecA-mediated cloning [35]. Clones generated by polymerase chain reaction (PCR) were verified by DNA sequencing.

#### Generation of FLAG-tagged DBP

The coding sequence for the FLAG-tag was added to DBP using overlap PCR. pRP2014 [15] was subjected to PCR in two separate reactions using the following synthetic oligonucleotides: 3016F1-GCAACGCGTCGCAGATAATGGCGAC and 3116R-CTTATCGTCGTCGTCCTTGTAATCGGGAGGCGGCGGCGACGGGGAC; 3116F-GATTACAAGGACGACGACGATAAGCGGGCGCCCCCAAAAAGC and 3016R2-CCAACGCGTTTAGCAGGTCGGGC. The two resulting DNA products were combined into a single PCR reaction and re-amplified with 3016F1 and 3016R2. The DNA product from this second amplification was digested with MluI and cloned into MluI-digested pGEM7, generating pRP3116. A 5520 bp BamHI/SpeI fragment from pRP2014 was also cloned into BamHI/SpeI-digested pBluescript, generating pRP3037. An 1821 bp MluI fragment from pRP3116 was then used to replace the MluI fragment in pRP3037, generating pRP3118. A 5544 bp BamHI/SpeI fragment from pRP3118 was cloned into pRP2014, generating pRP3120 which is an Ad5 genomic plasmid lacking E1 and E3, but containing a FLAG-tag on the DBP. pRP3120 was recombined with pRP2483 [36] to produce pRP3121, an E1/E3-deleted Ad5 genomic plasmid containing a monomeric red fluorescent protein (RFP) reporter gene [37] under the regulation of the cytomegalovirus (CMV) immediate early enhancer/promoter and bovine growth hormone polyadenylation sequence (BpA) replacing the E1 region, and the FLAG-tagged DBP. pRP3120 was also recombined with pXC1 [38] to generate pRP3122, an E1^+^/E3^-^ infectious plasmid containing the FLAG-tagged DBP. pRP3121 and pRP3122 were digested with PacI to liberate the inverted terminal repeats, transfected into 293 cells and recovered as viruses, which are designated AdE1^-^DBP-F and AdE1^+^DBP-F, respectively, in this study. In these viruses, the coding sequence for the FLAG-tag is located immediately before 23916 bp of the conventional human Ad5 genome (GenBank AC_000008). Please note that the E2 coding sequences runs right to left, so before 23916 bp actually places the FLAG-tag downstream of this position in the E2 open reading frame. The specific amino acid sequence of this region of DBP is 36-PPPP***DYKDDDDK***RAPP, where 36 refers to the position in the DBP of the first listed amino acid (in this case proline, P), the FLAG-tag is shown in italics and bold, and the native DBP sequence is in normal type.

#### Generation of FLAG-tagged TP

A 1615 bp MfeI fragment (8016 bp to 9631 bp of the Ad5 genome) containing a portion of the pTP coding sequence from pRP2014 was cloned into MfeI-digested pNEB193, generating pRP2982. The synthetic oligonucleotide CTTATCGTCGTCATCCTTGTA and its complement were ligated into EcoRV-digested pRP2982, generating pRP2988. A 1069 bp EagI/XbaI fragment from pRP2110a [39] was cloned into EagI/XbaI-digested pRP2982, which added an extra region of Ad genome homology and convenient restriction sites for the next step of cloning, and was designated pRP2994. A 2202 bp BsrGI/SnaBI fragment from pRP2982 was then cloned into BsrGI/SnaBI-digested pRP2110a, generating pRP2995. Finally, a 7124 bp XbaI fragment from pRP2995 was cloned into XbaI-digested pRP2014ΔXbaI, generating pRP2996, an Ad5 genomic plasmid lacking E1 and E3 but containing a FLAG-tag on the pTP. pRP2996 was recombined with pRP2483 to produce pRP2997, an E1/E3-deleted Ad5 genomic plasmid containing a CMV-RFP-BpA expression cassette replacing the E1 region and the FLAG-tagged pTP. pRP2996 was also recombined with pXC1 to obtain pRP3000, an E1^+^/E3^-^ infectious plasmid containing the FLAG-tagged pTP. pRP2997 and pRP3000 were digested with PacI to liberate the inverted terminal repeats, transfected into 293 cells and recovered as viruses, which are designated AdE1^-^TP-F and AdE1^+^TP-F, respectively. In these viruses, the coding sequence for the FLAG-tag is located immediately before 9201 bp of the Ad genome. The specific amino acid sequence of this region of TP is 463-EALG***DYKDDDDK***INES. Of note, the 467^th^ amino acid (D) is native to the TP protein and also constitutes the first residue of the FLAG-tag.

#### Generation of FLAG-tagged Pol

A 327 bp XhoI/NsiI fragment (XhoI at 8255 bp to NsiI at 8582 bp of the Ad5 genome) from pRP2014 was cloned into XhoI/NsiI-digested pGEM7, generating pRP2183. pRP2183 was amplified by PCR with synthetic oligonucleotides BRS100F-GCGAAGCTTCCGCCTGACCTGACGCCGC and BRS100R-GCGAAGCTTATCGTCGTCATCCTTGTAATCCTCCTGCAGGTTTACCTCGCATAGACG, which prime in opposite directions around the circular plasmid. The resulting DNA product was digested with HindIII and re-circularized, generating pBRS100. A 602 bp ApaI/XhoI fragment from pRP2110a was cloned into XhoI/ApaI digested pBRS100, generating pBRS101 and adding an extra region of Ad genome homology and convenient restriction sites for the next step of cloning. A 309 bp BsrGI/Sse8387I fragment from pBRS101 was cloned into pRP2110a, generating pBRS103. Lastly, a 7127 bp XbaI fragment from pBRS103 was cloned into XbaI digested pRP2014ΔXbaI, generating pBRS104, an Ad5 genomic plasmid lacking E1 and E3 but containing a FLAG-tag on the Pol. pBRS104 was recombined with pRP2483, generating pBRS105, an E1/E3-deleted Ad5 genomic plasmid containing a CMV-RFP-BpA expression cassette replacing the E1 region and the FLAG-tagged Pol. pBRS104 was also recombined with the pXC1, generating pBRS106, an E1^+^/E3^-^ infectious plasmid containing the FLAG-tagged Pol. pBRS105 and pBRS106 were digested with PacI to liberate the inverted terminal repeats, transfected into 293 cells and recovered as viruses, which are designated AdE1^-^Pol-F and AdE1^+^Pol-F, respectively. In these viruses, the coding sequence for the FLAG-tag is located immediately before nucleotide 8404 bp of the Ad genome. The specific amino acid sequence of this region of Pol is 127-NLQE***DYKDDDDK***LPPD.

### Cell lines and infections

293 (a kind gift from Dr. Frank Graham, Professor Emeritus, McMaster University) [40] and A549 cells (CCL-185, ATCC) were grown in Minimum Essential Medium (MEM, Sigma Aldrich) supplemented with 10% (v/v) Fetal Bovine Serum (FBS, Sigma Aldrich), 2 mM GlutaMAX (Invitrogen) and 1x antibiotic-antimycotic (Invitrogen). HepG2 cells (HB-8065, ATCC) were grown in Dulbecco’s Modified Eagle’s Medium (DMEM, Sigma-Aldrich) supplemented as above.

Viruses were propagated in 293 cells and purified by cesium chloride buoyant density centrifugation, using standard procedures [41]. Viral titers were determined by plaque assay on 293 cell monolayers and scored as the number of plaque-forming units (PFU) per milliliter of purified virus [41]. For all experiments, monolayers of A549 cells were infected at the indicated multiplicity of infection (MOI) with Ad5, AdE1^+^DBP-F, AdE1^-^DBP-F, AdE1^+^TP-F or AdE1^+^Pol-F diluted in phosphate-buffered saline (PBS, Sigma-Aldrich) for 1 hour at 37^°^C. Medium was then added to the cells and incubated in a humidified CO_2_ incubator at 37^°^C until the indicated time point. For the quantitative PCR (qPCR) experiments, the viral inoculum was removed after the 1-hour incubation and the monolayers were washed once with PBS before adding medium.

### Immunoblot analysis

To prepare protein samples, medium was removed from infected cells at the indicated time points, 2x protein sample buffer (62.5 mM Tris-HCl pH 6.8, 25% glycerol, 2% SDS, 0.01% bromophenol blue, 5% β-mercaptoethanol) was added, and the resulting crude protein extract was collected. The protein samples were boiled prior to separation by sodium dodecyl sulphate-polyacrylamide gel electrophoresis (SDS-PAGE). Proteins were transferred to a polyvinylidene fluoride (PVDF) membrane (Millipore) and the membrane was probed with monoclonal antibodies to Ad5 fiber (1/10,000 dilution; MS-1027-P0, Neomarkers), FLAG (M2, 1/5,000; 200-301-383S, Rockland), Ad5 E1A (1/5,000; ab31686, Abcam) or a monoclonal antibody to human vinculin (1/10,000; ab73412, Abcam). The monoclonal antibody against Ad5 DBP [32] was a kind gift from Dr. Arnold J. Levine (antibody 72K-B6, 1/500 dilution).

### Detection of 55-kDa TP within purified virions

For detection of the viral DNA-associated 55-kDa TP present within the virion, 3x10^10^ virus particles (VP) were diluted in 10 mM Tris pH 7.5 to a final volume of 25 μl and denatured for 10 min at 60°C. The samples were cooled to room temperature, prior to adding reaction buffer (final concentrations: 50 mM NaCl, 10 mM Tris-HCl pH 7.9, 10 mM MgCl_2_ 1 mM DTT) and 2 units of DNaseI (M0303S, New England BioLabs). After a 20-min incubation at 37°C, an equal volume of 2x protein sample buffer was added, the samples were boiled for 5 minutes, and analyzed by immunoblot as described above.

### Real-time quantitative PCR

Media from infected cells was removed at the indicated time points and the cells were incubated overnight at 37°C in SDS-proteinase K buffer (10 mM Tris-HCl pH 7.4, 10 mM EDTA, 1% SDS (w/v), 1 mg/ml proteinase K) [36]. Following phenol-chloroform extraction of the DNA, qPCR was conducted as previously described [41] using 200 ng of total genomic DNA per reaction. The following primers were used for the hexon region of the genome: 5’-CTT ACC CCC AAC GAG TTT GA and 5’-GGA GTA CAT GCG GTC CTT GT.

### Immunoprecipitation

Ten centimeter dishes of A549 cells were infected with AdE1^+^Pol-F or wildtype Ad5 at an MOI of 100, and lysed 24 hours later in modified radio immunoprecipitation assay (RIPA) extraction buffer (50 mM Tris pH 8.0, 100 mM NaCl, 1 mM EDTA, 1% glycerol, 1% NP-40, protease inhibitors). Immunoprecipitation was conducted as previously described [42], using 30 μl Protein G beads (1004D, Dynabeads) for preclearing and 2 μg FLAG antibody or IgG (sc-2025, Santa Cruz) as negative control. Twenty microliters of beads containing the protein-bound antibody was resuspended in 20 μl 2x protein sample buffer before analysis.

### Immunofluorescence

A549 cells were seeded on 1 cm rounded coverslips at 1/10^th^ of the confluency density in 12-well tissue-culture plates, and infected as described above with FLAG-tagged Ad constructs at MOIs varying from 10 to 50. Eighteen hours post infection (hpi), media was removed and the coverslips were washed once with cold PBS. The cells were fixed with 4% paraformaldehyde (pH 7.0) in PBS for 15 mins, rinsed with cold PBS, permeabilized with PBS containing 0.25% Triton X-100 (Thermo Fisher Scientific) for 10 min, and then washed three times with PBS for 5 min each. PBST supplemented with 1% BSA was added for 30 min to block nonspecific antibody binding, prior to overnight incubation at 4°C with the primary antibody diluted in the blocking solution. The next day, the primary antibody was decanted and the cover slips were washed three times as before. The coverslips were incubated for 1 hour at room temperature with secondary antibody, also diluted in the blocking solution. After secondary antibody removal and three washes with PBS in the dark, the cells were incubated with Hoechst 3342 (1 μg/ml, H3570, Life Technologies) for 1 min. Lastly, the coverslips were rinsed with PBS and placed on glass slides using Dako Fluorescent Mounting Medium (Agilent Technologies). The following antibodies were used: mouse monoclonal anti-FLAG antibody (M2, 1/1,000; 200-301-383S, Rockland) and Alexa Fluor 594 anti-mouse IgG (1/5,000; A21203, Thermo Fisher Scientific). Fluorescent images were obtained with a Plan-APOCHROMAT 63x/1.4 oil-immersion Ph3 objective on a Zeiss Axio Observer.Z1 microscope equipped with an Axiocam MRC camera and ZEN 2 software for image processing.

## Results

### Insertion of a FLAG-epitope tag into E2-encoded proteins

The N-terminal region (amino acids 1–173) of the Ad5 DBP is relatively weakly conserved, and is dispensable for ssDNA binding activity [43]. In contrast, the C-terminal region (amino acids 174–529) is highly conserved and is directly involved in DNA binding [44]. Indeed, the cooperative binding of DBP to ssDNA is mostly lost when the very distal C-terminal 17 amino acids of DBP are removed [43]. Vos *et al*. used insertion mutagenesis studies to identify regions of the DBP that were tolerant for insertion of small peptides [45]. In this approach, small synthetic oligonucleotides (12 bp, corresponding to 4 amino acids) were cloned into various positions in the E2A coding region, and protein function and/or ability to recover the virus was evaluated. If protein function was retained and virus was recovered, the specific region of the protein was tolerant of insertions, suggesting that the region was perhaps not crucial for protein function. However, if no virus was recovered, the region was deemed not tolerant of mutation and likely crucial for function of the protein. Vos *et al*. identified the region between amino acids 39 and 41 of DBP as tolerant for insertion of small peptides with no loss of virus replicative ability [45]. Thus, to create our FLAG-tagged DBP construct, we placed the tag following amino acid 39. This virus, designated AdE1^+^DBP-F, was easily rescued and showed similar fitness to wildtype Ad5 (Fig 1B and 1C). An E1-deleted version was also generated, designated AdE1^-^DBP-F.

Studies by Roovers *et al*. [46] used insertion mutagenesis to identify crucial regions of the E2B-encoded TP and Pol. We used this study to identify regions of the E2B-encoded proteins that would likely accept the FLAG epitope tag. For pTP, Roovers *et al*. identified two insertion sites, ins318 and ins467, that retained full activity during DNA replication [46]. We wished to place an epitope tag within pTP in such a way that the tag would be retained in the mature form of the protein after cleavage by the Ad-encoded protease. pTP is initially cleaved by the Ad protease between residues 175–176 or 183–184, generating an intermediate TP (iTP), followed by cleavage between residues 349–350, generating the mature TP [47]. TP is covalently attached to the viral DNA at Ser580 [48]. Thus, we chose to insert the FLAG-tag after amino acid 466 (see Materials and Methods). This virus, designated AdE1^+^TP-F, was rescued and grew similar to wildtype Ad5 (Fig 1B and 1C). An E1-deleted version of this virus was also generated, designated AdE1^-^TP-F.

The insertion mutagenesis studies by Roovers *et al*. also identified two insertion sites within the Ad5 Pol that showed near wildtype polymerase activity: ins130 and ins243. However, only ins130 also retained a significant ability to form the initiation complex through catalyzing the covalent addition of dCMP to pTP [49]. Insertion of the FLAG at amino acid position 130 within the Pol gene resulted in a viable virus, designated AdE1^+^Pol-FLAG, that replicated its DNA and expressed the late fiber gene at a level similar to wildtype Ad5 (Fig 1B and 1C). The various viruses are summarized in Table 1.

**Table 1:** List of Ad constructs used in this study.

Ad construct^a^	Modifications to E1 region	Modifications to E2 region	Location of FLAG-tag^b^
Ad5	N/A^c^	N/A	N/A
AdE1^+^DBP-F	N/A	DBP-FLAG	39
AdE1^-^DBP-F	ΔE1, CMV-RFP	DBP-FLAG	39
AdE1^+^TP-F	N/A	TP-FLAG	466
AdE1^-^TP-F	ΔE1, CMV-RFP	TP-FLAG	466
AdE1^+^Pol-F	N/A	Pol-FLAG	130
AdE1^-^Pol-F	ΔE1, CMV-RFP	Pol-FLAG	130

^a^Ad5 is wildtype virus, and retains the native E1 and E3 coding regions. AdE1^+^DBP-F, AdE1^+^TP-F, and AdE1^+^Pol-F retain the native E1 coding region, but lack the E3 coding region. AdE1^-^DBP-F and AdE1^**-**^TP-F lack the E1A, E1B and the E3 coding regions.

^b^The last column provides the number of amino acid residues after which the FLAG-tag was inserted in the protein sequence.

^c^Not applicable (N/A).

### Virus growth is unaffected by the presence of the FLAG-tag within the E2 region proteins

To determine whether insertion of the FLAG-tag affected DNA replication or late gene expression of the viral constructs, monolayers of A549 cells were infected at an MOI of 10 with wildtype Ad5, AdE1^+^DBP-F, AdE1^+^TP-F or AdE1^+^Pol-F for 18 or 24 hours. The infected cells were then lysed in appropriate buffers for protein and DNA isolation. Analysis of Ad fiber expression following SDS-PAGE and immunoblot analysis showed no significant difference in late gene expression from the FLAG-tagged viruses compared to Ad5 (Fig 1B). Viral DNA copy numbers in the infected cells were also examined by qPCR with primers specific to the Ad hexon region, and DNA replication levels of these viruses appear to be similar to Ad5 as well (Fig 1C). All of the FLAG-tagged Ad constructs had production titers above 10^9^ pfu/ml (as determined by plaque assay on 293 cell monolayers), suggesting that presence of the FLAG-tags does not impact virus infectivity or growth.

### Detection of the FLAG-tagged E2A and E2B proteins

**Fig 2:**
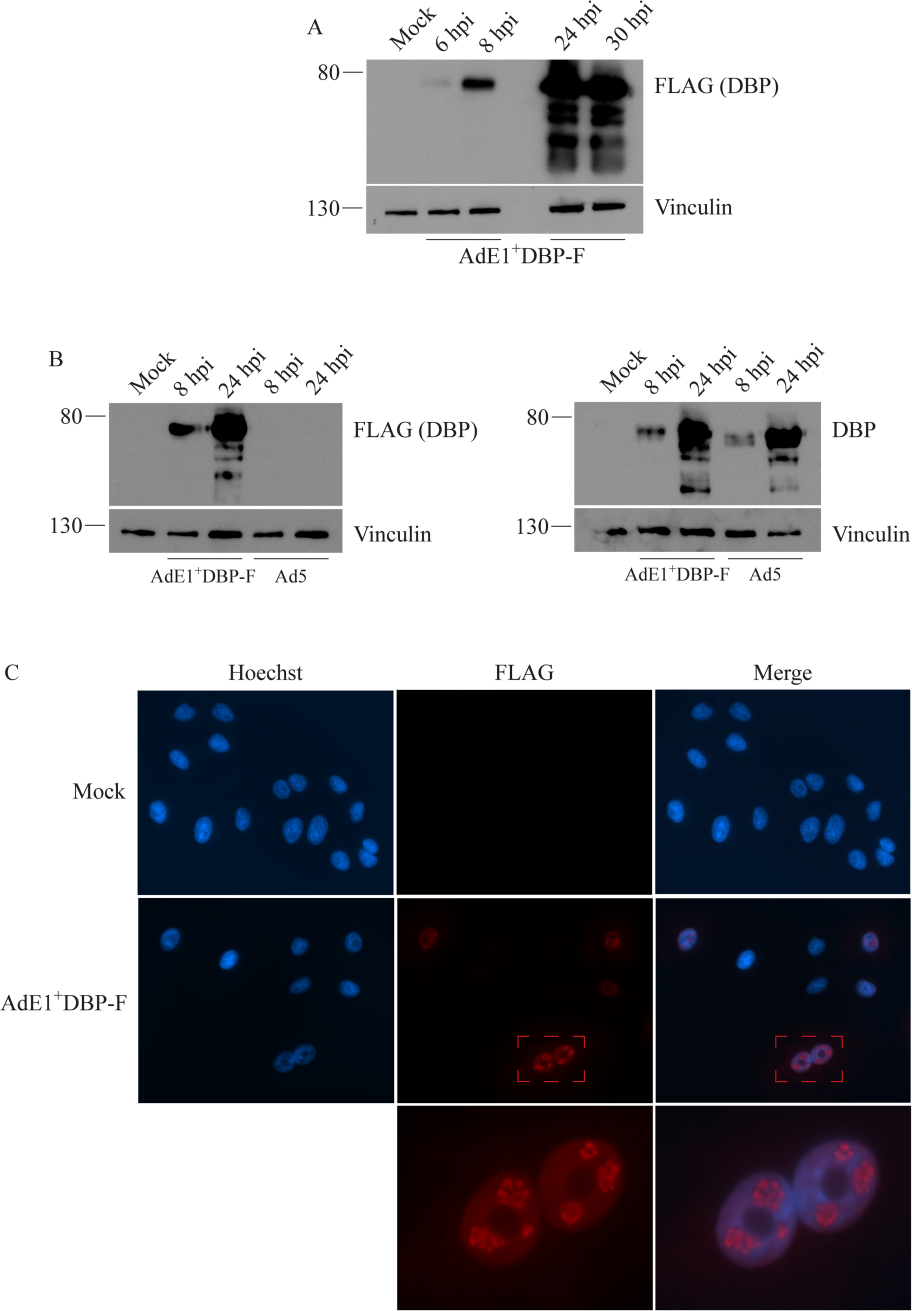
Expression of the E2A-encoded, FLAG-tagged DBP from AdE1^+^DBP-F. (A) A549 cells were infected with AdE1^+^DBP-F at an MOI of 50, and crude cell lysates were analyzed for FLAG-tagged DBP expression at varying times post-infection. DBP was readily detected via immunoblotting in cell lysates during both early and late infection. (B) An experiment similar to that in panel A was conducted with Ad5 (MOI 50) in addition to AdE1^+^DBP-F. The lysates were analyzed in duplicate immunoblots to detect DBP using either an anti-FLAG or an anti-DBP antibody. (C) Immunofluorescence analysis of FLAG-tagged DBP expression. A549 cells were plated at 1/10^th^ of the confluency density and infected at an MOI of 10. At 18 hpi, distinct Ad replication foci within the host cell nuclei were visible through immunofluorescence imaging for FLAG-DBP.

We next determined whether the presence of the FLAG-tag on the various E2-encoded proteins could allow them to be detected by immunoblot and immunofluorescence. To characterize expression of the DBP, A549 cells were infected with AdE1^+^DBP-F at an MOI of 50, and crude protein extracts were prepared at varying times post-infection. As shown in Fig 2A, the 72-kDa FLAG-tagged DBP was detected as early as 6 hpi. At late infection times (24 and 30 hpi), the quantity of full-length DBP within the infected cell was very high, and several smaller FLAG-tagged protein species were also present. These smaller species may be the result of premature translation termination, proteolytic degradation, or perhaps alternative splicing events within the E2A transcript [50]. Using a lower MOI of 10, the FLAG-tagged DBP was easily detected by immunoblot at a slightly later time point of 8 hpi (data not shown). We next compared the level of DBP expression from AdE1^+^DBP-F with wildtype Ad5 to determine whether presence of the FLAG tag altered expression. A549 cells were infected with the two viruses at an MOI of 50, protein samples were collected 8 and 24 hpi, and DBP expression was evaluated by immunoblot using an antibody to native DBP. As shown in Fig 2B, presence of the FLAG-tag did not affect the level of expression of DBP from the virus. The FLAG-tag also allowed us to visualize DBP by immunofluorescence. Ad replication foci within infected cell nuclei were clearly observed at an MOI of 10 using the antibody directed against the FLAG-tag on the DBP (Fig 2C). Thus, the FLAG-tagged DBP is readily detectable by immunoblot and immunofluorescence in infected cells.

**Fig 3:**
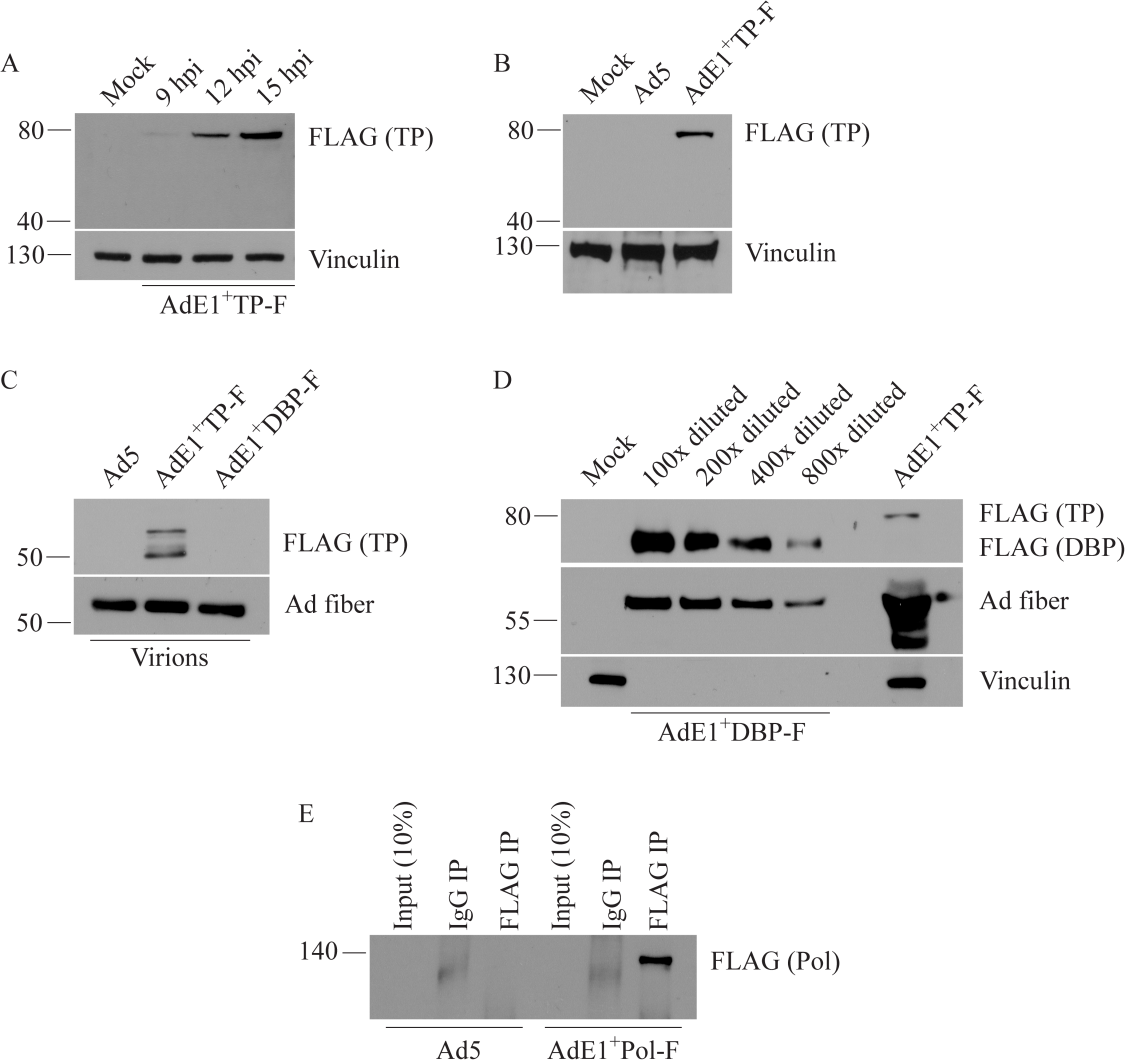
Expression of the E2B-encoded, FLAG-tagged TP and Pol. (A) A549 cells were infected with AdE1^+^TP-F at an MOI of 50 and expression of the 80-kDa pTP was observed as early as 9 hpi. (B) Even at a later time point of 24 hpi, only the 80-kDa pTP was detected in infected cell lysates, but not the 55-kDa mature protein. (C) Ad5, AdE1^+^DBP-F or AdE1^+^TP-F (3x10^10^ virus particles) were heat-denatured and DNase-treated. The Ad DNA-associated 55-kDa TP was readily detected by immunoblot. (D) Lysate of cells infected for 24 hrs with AdE1^+^DBP-F was serially diluted 100- to 800-fold. DBP expression in the diluted samples and TP expression in the undiluted AdE1^+^TP-F-infected cell lysate were analyzed by immunoblot to compare expression levels of the two proteins. Undiluted sample showed equal intensity when probed for vinculin (data not shown). (E) A549 cells were infected with Ad5 or AdE1^+^DBP-F at an MOI of 100. Immunoprecipitation was conducted 24 hrs later, followed by SDS-PAGE and immunoblot analysis of the samples. When probed with the anti-FLAG antibody, a single band corresponding to the 140-kDa Ad Pol was clearly visible in lysates of cells infected with AdE1^+^DBP-F.

Unlike the abundantly expressed DBP, biochemical studies on Ad Pol and pTP have been difficult to conduct due to low expression levels of these two proteins, and existence of these proteins as a pTP-Pol complex in infected cells [11,34]. Consistently, we were not able to detect the presence of pTP in cell lysates at 6 or 8 hpi using an MOI of 50 (data not shown), but the FLAG-tagged pTP could be detected at low levels from 9 hpi (Fig 3A). Since both DBP and pTP contain an identical epitope tag, and were thus detected using the same antibody against FLAG, our inability to detect pTP at the earlier time points supports the idea that pTP is expressed at a significantly lower level than DBP in the infected cell (with the caveat that the differences in amino acid composition surrounding the FLAG tag in the two proteins may also influence the efficiency of antibody binding). We were also unable to visualize the FLAG-tagged TP by immunofluorescence (data not shown), once again suggesting that the expression levels of pTP from E2B are much lower than DBP from E2A. Only the 80-kDa pTP was observed in infected cells, even at the very late time point of 24 (Fig 3B) or 48 hpi (data not shown). We did not observe the mature 55-kDa protein in infected cell lysates, which was somewhat surprising considering that significant numbers of mature virions had likely accumulated in the nucleus of the infected cells by 24 hpi. It is possible that the 55-kDa TP remained attached to the newly-synthesized viral genomes and did not enter the gel, but remained in the sample loading wells instead. Following digestion with DNaseI, the proteolytically-processed 55-kDa mature TP associated with the viral DNA was readily detected by immunoblot analysis of purified virions (Fig 3C). Interestingly, we observed two prominent protein bands from the purified virions (Fig 3C), which may represent cleavage of the iTP by the Ad protease at positions 317 and 349 [51,52,53]. However, cleavage at position 317 has not been observed experimentally, although there is a type 2 consensus cleavage site for the Ad protease present at that position [51]. Alternatively, the two TP species present in mature Ad virions may reflect a difference in post-translational modification of the mature 55-kDa TP [54,55], or perhaps a small number of viral nucleotides remaining attached to the DNase-treated sample. Regardless, pTP and TP are clearly detectable with the FLAG antibody in protein lysates from infected cells and purified virions, respectively.

The results described above indirectly suggest that pTP is expressed at a much lower level in infected cells compared to DBP. To directly compare the relative level of pTP and DBP expression, we analyzed serially diluted lysates from cells infected with AdE1^+^DBP-F and undiluted lysate from cells infected with AdE1^+^TP-F. Comparison of the signal obtained for pTP with the dilution series of DBP indicates that DBP is expressed at an approximately 800-fold higher level than TP under the conditions examined (A549 cells, MOI of 50, 24 hpi) (Fig 3D).

Repeated attempts to detect the FLAG-tagged Ad DNA Pol in crude protein lysates by standard immunoblot, even at as high an MOI as 100, were unsuccessful (data not shown), suggesting that Pol is expressed at very low levels in infected cells. However, using a standard immunoprecipitation assay, we could detect a 140-kDa species representing the full-length Pol (Fig 3E). As might be expected, attempts to detect Pol through immunofluorescence of infected cells were unsuccessful (data not shown), once again suggesting that expression of this E2B-encoded Pol protein is extremely low in infected cells.

### E2 proteins are expressed at a very low level from E1-deleted Ad vectors

Previous studies have suggested that E1-deleted Ad vectors can replicate in many cell types, particularly transformed cells [26]. For example, using an assay in which infecting viral DNA was specifically methylated to prevent cleavage by XhoI restriction endonuclease, and newly-replicated viral DNA became sensitive to cleavage due to absence of this methylation, Nelson and Kay estimated that infection of A549 cells with an E1-deleted virus at an MOI of 100 resulted in DNA replication at a level of 84% of an E1^+^ virus at 72 hpi [27]. Varying levels of replication were observed in almost all cell lines tested in the study, suggesting that E1-deleted viruses could undergo a surprisingly high level of replication in “non-permissive” cells. Since E2 proteins are required for viral DNA replication, and full activation of the E2 promoter requires E1A [30], it has been suggested that certain cellular factors may be able to compensate for lack of E1A in E1-deleted viruses, resulting in expression of the E2 proteins. Several cellular factors have been implicated in complementing the loss of E1A, including in the A549 cell line used in our studies [26,56]. In these studies, viral gene expression and replication of the E1-deleted vectors were enhanced with increasing MOI, suggesting a cooperative effect in which each additional genome contributed replicative proteins to enhance the level of replication. Consequently, there may be a threshold of replicative proteins required to initiate Ad DNA replication [26,27,56].

**Fig 4:**
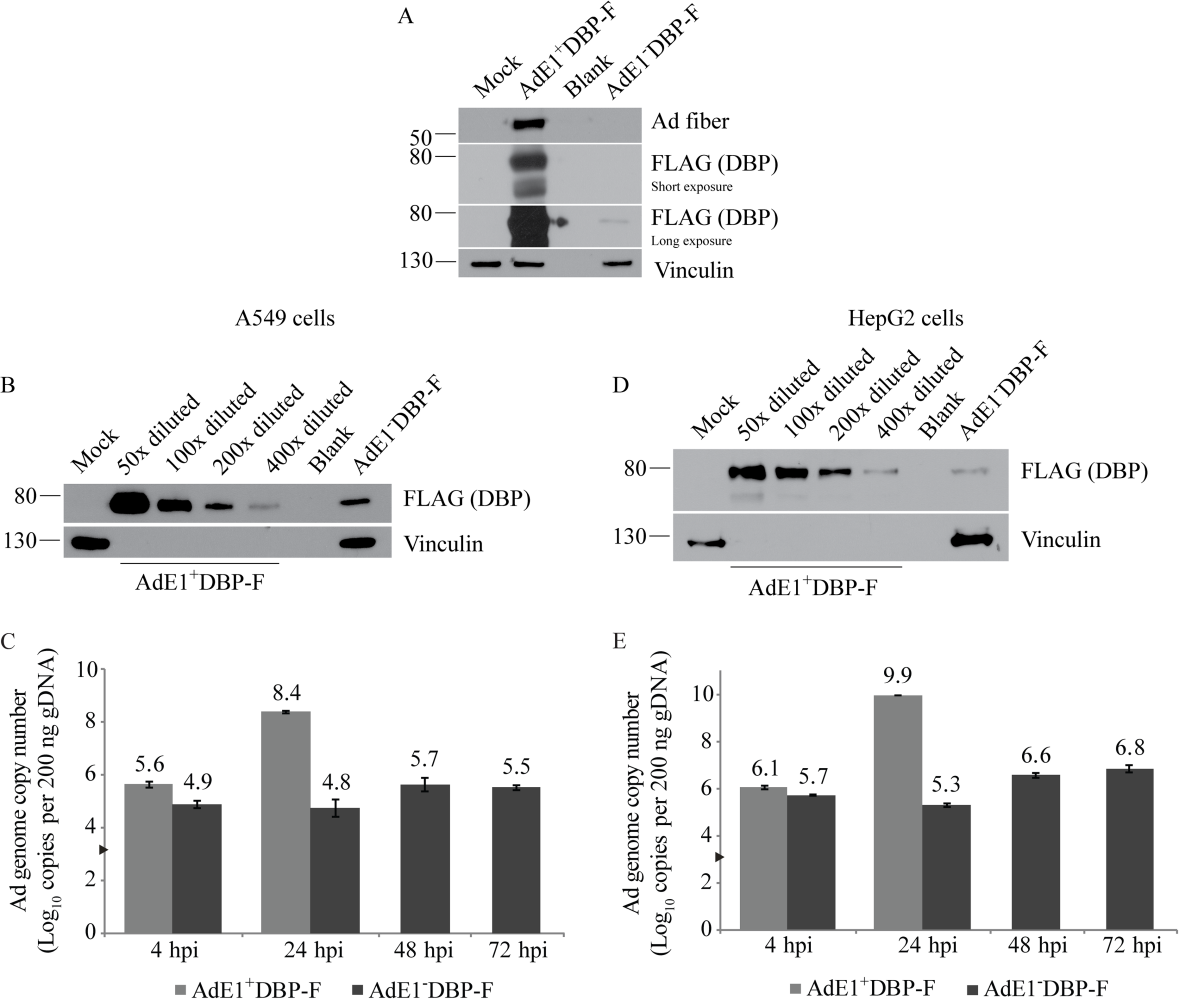
Removal of the E1 region results in a dramatic reduction in E2 gene expression and DNA replication in A549 and HepG2 cells. (A) A549 cells were infected at an MOI of 10 with AdE1^+^DBP-F or AdE1^-^DBP-F. Cell lysates isolated at 24 hpi were analyzed for fiber and DBP expression via immunoblot. DBP expressed from the AdE1^-^DBP-F was only observed at high exposure. (B) The AdE1^+^DBP-F-infected sample used in panel A (MOI 10, 24 hpi) was diluted 40- to 500-fold in 2x protein loading buffer. FLAG-DBP expression in undiluted AdE1^-^DBP-F-infected cell lysate and diluted AdE1^+^DBP-F-infected cell lysates were detected by immunoblot to compare expression from the two constructs. (C) A549 cells were infected with AdE1^+^DBP-F for 4–24 hours or AdE1^-^DBP-F for 4–72 hours. DNA was harvested and qPCR was performed as described above to determine the copy numbers of viral DNA per 200 ng DNA isolated from infected cells. The mean of three independent experiments is shown and the error bars represent SD. The arrowhead on the y-axis indicates the limit of detection for the assay, and represents the background copy number calculated in uninfected cells. (D) Lysates of HepG2 cells infected with AdE1^+^DBP-F (MOI 10, 24 hpi) were diluted as described in panel B to compare DBP expression from the E1^+^ and E1^-^ viruses in this cell line. Undiluted sample showed equal intensity when probed for vinculin (data not shown). (E) HepG2 cells were infected and qPCR was conducted as explained for A549 cells in panel C.

The placement of a FLAG-tag on the E2 proteins provided us with unique reagents to examine the relative level of E2 expression from E1-competent and E1-deficient virus in A549 cells. Given the low expression levels of pTP and Pol within the infected cells, we used the AdE1^+^DBP-F and the AdE1^-^DBP-F constructs to take advantage of the higher DBP expression levels, and thus greater sensitivity. A549 cells were infected at an MOI of 10 with AdE1^+^DBP-F or AdE1^-^DBP-F and 24 hr later, crude protein extracts were prepared and analyzed for DBP and fiber expression by immunoblot. As shown in Fig 4A, we observed a very strong DBP signal for AdE1^+^DBP-F, but the level of DBP expression from AdE1^-^DBP-F was very low and only visible on overexposure of the blot. Similarly, although expression of the late fiber protein, which is only expressed following DNA replication, was easily detected in cells infected with AdE1^+^DBP-F, no fiber expression was observed in cells infected with AdE1^-^DBP-F. To better gauge the relative level of DBP expression between the two viruses, we analyzed a dilution series of lysate from cells infected with AdE1^+^DBP-F relative to undiluted AdE1^-^DBP-F cell lysate. AdE1^-^DBP-F expressed the E2A-encoded DBP at a level approximately 1/200^th^ that of the E1^+^ virus (Fig 4B) under these conditions (MOI of 10, 24 hpi). We also analyzed DNA replication directly by qPCR at 4, 24, 48 and 72 hpi (for AdE1^+^DBP-F, samples were isolated only at 4 and 24 hpi). Although cells infected with both viruses showed comparable levels of viral DNA at 4 hpi (prior to initiation of DNA replication), there was a ~4-log increase in viral genome copy number at 24 hpi for AdE1^+^DBP-F, but no change for AdE1^-^DBP-F (Fig 4C). Even at the later time points of 48 and 72 hpi, there was only a very modest increase in viral genome copy number in cells infected with AdE1^-^DBP-F, amounting to a ~5-fold increase in genome copy number within the cells between 24 and 72 hours. Thus, at a relatively low MOI of 10, AdE1^-^DBP-F does express some DBP, but the level is significantly lower than that from the E1^+^ virus, and is not sufficient to allow efficient viral DNA replication and subsequent late gene expression. Similar experiments were conducted in human HepG2 cells and the observations were consistent with those in A549 cells (Fig 4D and 4E). DBP expression from the AdE1^-^DBP-F was 1/400^th^ of that from AdE1^+^DBP-F at an MOI of 10 for samples isolated at 24 hpi (Fig 4D); although viral DNA replication was slightly higher for both viruses in HepG2 cells compared to A549 cells, only a small increase in genome copy number was observed for AdE1^-^DBP-F between 24 and 72 hours (Fig 4E). Taken together, these results suggest that in the absence of E1 proteins, Ad vectors undergo very limited E2 expression and DNA replication in infected cells.

### E1-deficient Ad cooperate to enhance virus replication in an additive, but not synergistic, manner

**Fig 5:**
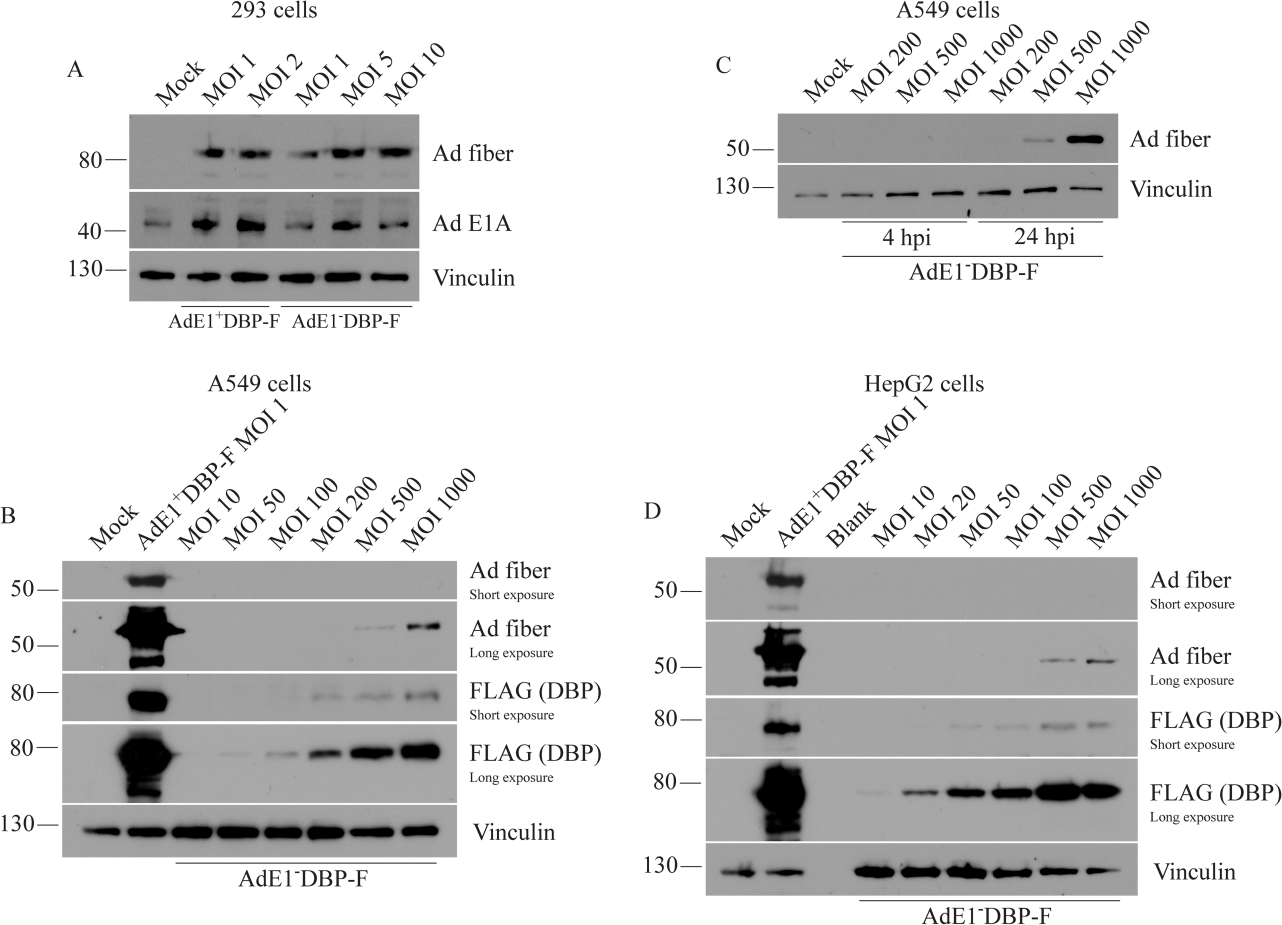
Late gene expression from the E1-deficient FLAG-tagged DBP construct is observed at very high MOIs. (A) 293 cells were infected for 24 hours with the indicated MOI of AdE1^+^DBP-F and AdE1^-^DBP-F. Cell lysates were separated by SDS-PAGE and analyzed for fiber expression via immunoblot. (B) A549 cells were infected for 24 hours with AdE1^+^DBP-F at an MOI of 1 or increasing MOI of AdE1^-^DBP-F to determine the level of DBP necessary to achieve fiber expression, which is indicative of AdE1^-^DBP-F replication. Immunoblot analysis of fiber and DBP expression was conducted. (C) Fiber expression from AdE1^-^DBP-F was examined at 4 and 24 hpi in A549 cells. (D) An experiment similar to that in panel A was conducted in the HepG2 cell line.

We next examined the ability of the E1-deficient virus to synergize in high-MOI infections and enhance virus replication in non-permissive cells (i.e. those not complementing the E1-deficiency). If each virus contributes a small amount of E2 proteins, then with increasing MOI, the cumulative expression could reach the threshold required for efficient replication. Consequently, with increasing MOI, we should observe a non-linear increase in replication and late gene expression. We first examined whether the E1-deleted and the E1^+^ virus had similar abilities to replicate under conditions in which E1 was supplied (i.e. in the E1-complementing 293 cell line). 293 cells were infected at low MOIs with AdE1^+^DBP-F or AdE1^-^DBP-F, crude proteins samples were isolated 24 later, and evaluated for the level of fiber protein (a surrogate marker for viral DNA replication). As shown in Fig 5A, both viruses replicated well, and we did not observe a significant difference in fiber expression between the two constructs. Thus, E1-deleted viruses display wildtype levels of gene expression and replication when E1 is provided *in trans*. We next examined replication for the E1-deleted virus in A549 cells as a function of MOI. We saw an approximately linear increase in expression of DBP from AdE1^-^DBP-F between an MOI of 50 to 1000 (Fig 5B); however, the level of DBP never reached that produced by the E1^+^ version of the tagged virus applied to cells at an MOI of 1. We could detect a low level of fiber expression from AdE1^-^DBP-F starting at an MOI of 500, suggesting that sufficient DBP, and presumably also the replicative proteins encoded by E2B, was produced to allow for replication of the genome and expression of fiber (Fig 5B). Similar to our observation for DBP, even at an MOI as high as 1000, AdE1^-^DBP-F did not express fiber at a level comparable to the low-MOI AdE1^+^DBP-F infection. To confirm that the fiber signal observed for AdE1^-^DBP-F at 24 hpi was due to *de novo* gene expression and not simply from the infecting capsids, we conducted a time-course of expression from AdE1^-^DBP-F. As expected, we did not observe fiber signal at 4 hpi at any MOI, but fiber was detectable at the 24-hour time point for infections using only the very high MOIs (Fig 5C). Comparable results were obtained when examining the relationship between MOI, DBP and fiber expression in HepG2 cells (Fig 5D). Taken together, these results suggest that E1-deficient viruses can cooperate at high MOI to provide sufficient replicative proteins to promote DNA replication and late gene expression, but this effect does not appear to be synergistic–at least not at the range of MOI we have tested.

## Discussion

In this study, we have created Ad constructs containing a FLAG-tag in each of the three E2 protein coding regions (Table 1). Determination of the locations for the FLAG-tag insertion was aided by previous biochemical and linker-insertion mutagenesis studies that identified crucial regions of these proteins, and also regions that were tolerant for insertions of small peptides. We showed that all three proteins could be detected in Ad-infected cells by immunoblot (Figs 2 and 3); however, the low quantities of Pol necessitated the use of immunoprecipitation to concentrate the protein to a detectable level. The presence of the FLAG at amino acid position 467 of pTP allowed the tag to be retained in both the immature and mature version of the protein. Indeed, we easily observed only the 80-kDa immature protein in the infected cell lysates, and the cleaved 55-kDa version in mature virions (Fig 3). Since FLAG-directed reagents are readily commercially available, these E2-tagged viral vectors may be an important resource, permitting renewed investigation of these viral proteins.

Using the FLAG-tagged Ad constructs, we showed that during low MOI infections, the level of DBP expressed from E1-deleted Ad in A549 and HepG2 cells is very low and does not support efficient replication (Fig 4). These observations highlight the importance of E1A for full activation of the E2 promoter through both direct and indirect mechanisms. Although the Ad-encoded E4-ORF6/7 fusion protein can activate the E2 promoter to some extent, it is simply not efficient in the absence of E1A [30]. Alternatively, since E1A transactivates the E4 promoter, the reduced levels of E4-ORF6/7 may contribute to low E2 promoter activity. In either case, the cellular factors that have been implicated in complementing the loss E1A [56], leading to activation of the E2 promoter, are likely not as efficient as the native viral protein. Simply put, in the absence of E1A, insufficient E2 proteins are produced to support efficient viral DNA replication. However, previous studies have reported that under some conditions, E1-deleted viruses can replicate at a significant level in non-permissive cells, but this typically requires a high MOI [26,27,56]. In our study, we showed that even at an MOI as high as 1000, an E1-deleted vector expressed DBP at a level that was still substantially lower than a comparable E1^+^ virus at an MOI of 1 (Fig 5). Nevertheless, we did detect fiber expression from the E1-deficient virus at an MOI of 500 and above, suggesting that viral DNA replication had occurred (Fig 5). It is possible that at higher MOIs, the additive effect of each genome enhances the pool of available viral replicative proteins to the point where viral replication can occur. However, aside from expression of the E2 replicative genes, loss of E1A also impacts many other aspects of virus gene expression and replication which likely cumulatively compromise efficient virus replication. For example, since E1A has been shown to interact with several cellular transcription factors (*e*.*g*. SP1) to directly activate expression from the MLP [57], E1A deletion also directly reduces late gene transcription. Furthermore, E1A interacts with several cellular epigenetic regulatory proteins to cause a global change in epigenetic status of many genes within the host cell, which alters the cellular microenvironment to promote efficient virus replication [58,59]. E1A may also be involved in the epigenetic restructuring of the Ad genome early during infection [60]. This wide-ranging epigenetic reprogramming of cellular gene expression would not occur in cells infected with an E1-deleted Ad.

Taken together, our work underscores the crucial role of E1A in orchestrating and modulating various cellular and viral processes to achieve efficient Ad replication. However, the results also clearly show that MOI-dependent effects on replication of E1-deleted Ad is an important consideration when using Ad vectors for gene delivery. Attempts to increase transgene expression through use of high MOI infections may have the undesired effect of enabling Ad replication, which could ultimately compromise the integrity of the study.
